# Developing and testing a protocol using a common data model for federated collection and analysis of national perinatal health indicators in Europe

**DOI:** 10.12688/openreseurope.15701.1

**Published:** 2023-04-12

**Authors:** Jennifer Zeitlin, Marianne Philibert, Francisco Estupiñán-Romero, Marzia Loghi, Luule Sakkeus, Željka Draušnik, Adela Recio Alcaide, Mélanie Durox, Jan Cap, Jelena Dimnjakovic, Janis Misins, Enrique Bernal Delgado, Martin Thissen, Mika Gissler, Euro-Peristat Research Group

**Affiliations:** 1 Université Paris Cité, INRAE, Centre for Research in Epidemiology and StatisticS (CRESS), Obstetrical, Perinatal and Pediatric Epidemiology Research Team, EPOPé, INSERM, Paris, 75004, France; 2 Data Sciences for Health Services and Policy Research, Institute for Health Sciences in Aragon (IACS), Zaragoza, Spain; 3 Directorate for Social Statistics and Welfare, Italian Statistical Institute (ISTAT), Rome, Italy; 4 Estonian Institute for Population Studies, Tallin University, Tallin, Estonia; 5 Croatian Institute of Public Health, Zagreb, Croatia; 6 University of Alcala, Madrid, Spain; 7 National Health Information Center, Bratislava, Slovakia; 8 Centre for Disease Prevention and Control of Latvia, Riga, Latvia; 9 Department of Epidemiology and Health Monitoring, Robert Koch Institute, Berlin, Germany; 10 Department of Knowledge Brokers, THL Finnish Institute for Health and Welfare, Helsinki, Finland; 11 Department of Molecular Medicine and Surgery, Karolinska Institute, Stockholm, Sweden

**Keywords:** Birth data, federated analysis, newborn, maternal, perinatal, caesarean delivery, population health indicators, national statistics

## Abstract

**Context:** International comparisons of the health of mothers and babies provide essential benchmarks for guiding health practice and policy, but statistics are not routinely compiled in a comparable way. These data are especially critical during health emergencies, such as the coronavirus disease (COVID-19) pandemic. The Population Health Information Research Infrastructure (PHIRI) project aimed to promote the exchange of population data in Europe and included a Use Case on perinatal health.

**Objective**: To develop and test a protocol for federated analysis of population birth data in Europe.

**Methods:** The Euro-Peristat network with participants from 31 countries developed a Common Data Model (CDM) and R scripts to exchange and analyse aggregated data on perinatal indicators. Building on recommended Euro-Peristat indicators, complemented by a three-round consensus process, the network specified variables for a CDM and common outputs. The protocol was tested using routine birth data for 2015 to 2020; a survey was conducted assessing data provider experiences and opinions.

**Results:** The CDM included 17 core data items for the testing phase and 18 for a future expanded phase. 28 countries and the four UK nations created individual person-level databases and ran R scripts to produce anonymous aggregate tables. Seven had all core items, 17 had 13-16, while eight had ≤12. Limitations were not having all items in the same database, required for this protocol. Infant death and mode of birth were most frequently missing. Countries took from under a day to several weeks to set up the CDM, after which the protocol was easy and quick to use.

**Conclusion:** This open-source protocol enables rapid production and analysis of perinatal indicators and constitutes a roadmap for a sustainable European information system. It also provides minimum standards for improving national data systems and can be used in other countries to facilitate comparison of perinatal indicators.

## Plain language summary

Comparisons of indicators of the health of mothers and babies in European countries play a key role in the evaluation of health policies and health care practices. Even though most countries in Europe produce routine statistics on subjects such as stillbirth, neonatal mortality, and preterm birth, this is not done in a consistent way using common definitions which could enable the construction of a common database. During the coronavirus disease (COVID-19) pandemic, the difficulty of obtaining comparable data prevented the assessment of how the pandemic was affecting pregnancy outcomes. This study describes how the Euro-Peristat network worked within the European Population Health Information Research Infrastructure (PHIRI) project to develop new procedures to improve data on births available from national statistical systems. Based on previous work and consultation with members, the network selected data items and created a common data model with definitions, codes, and formats for the data. The common data model included 17 core data items for this testing phase and 18 for a future expanded phase. This model was used to run the same statistical programme in 28 countries and the four UK nations to produce aggregate tables that were then combined and analysed. Seven countries had all core items, 17 had 13-16, while 8 had <12. The most frequently missing data items were for infant death and mode of birth. Depending on how their data were organised, it took each data provider from under a day to over several weeks, to set up the database and test the protocol. Once the model was set up, partners found it quick and easy to use. Our study shows that these procedures for making harmonised data available are feasible and, if implemented more widely, could enable rapid production of important indicators of the health of mothers and babies.

## Introduction

The SARS-CoV-2 pandemic has drawn attention to both the importance of and the barriers to timely analysis of national population health data at a European level ^1^. While surveillance systems to track coronavirus disease (COVID-19) infections and hospital admissions were established ^2^, multiple questions about the direct effects and more crucially the indirect effects of the epidemic on population health and wellbeing remain unanswered. In particular, it is difficult to obtain reliable data about pregnant women and babies. Although these are generally healthy, low-risk populations, they are highly vulnerable to infectious disease outbreaks ^3^. Big populations are needed for accurate ascertainment of the most severe outcomes, such as stillbirth and infant death as they occur in only around 3 to 6 per 1000 births ^4^. A population approach is also essential because of the major impact of social factors, as observed in the recent pandemic ^5, 6^. Further, as disruptions to health care can change where and how antenatal and maternity care are provided, this can complicate surveillance over time in centre-based studies.

The COVID-19 pandemic compounded the pre-existing difficulties accessing comparable and timely data and the ways these limit the monitoring of the health of pregnant women and their babies and the effectiveness of perinatal health policies. Despite major declines in perinatal mortality and morbidity over the past 50 years, the health burden associated with pregnancy complications remains a public health priority in Europe where about 40,000 stillbirths and infant deaths still occur every year ^4^. Preterm birth and intrauterine growth restriction affect over 400,000 children. These are associated with morbidity at birth and affected children have higher risks of neurodevelopmental, respiratory and metabolic problems in childhood and adulthood than children born at term or with appropriate growth ^7, 8^. The considerable improvements in perinatal health witnessed over the past century have slowed and perinatal mortality may even be increasing in some countries ^4, 9^. The ability to measure these trends and provide benchmarks to countries with similar standards of living and health care provision is essential for guiding and evaluating perinatal policy in Europe.

While some perinatal health indicators are included in routine international databases such as the World Health Organization and Eurostat, they make no allowances for differences in definition and this compromises comparability ^10, 11^. In addition they do not include key indicators, such as the preterm birth rate or mortality for preterm babies. Euro-Peristat, an EU funded network of epidemiologists, public health specialists, statisticians and clinicians with experience in the use of routine population birth data, has published more comprehensive data, collected using a common protocol in a series of reports, but there is no sustainable system for compiling these data routinely.

The aim of this study, conducted by the Euro-Peristat network as part of the European H2020 Population Health Information Research Infrastructure (PHIRI) project, is to set up and test an open-source data collection protocol to facilitate exchange and federated analysis of comparable data about the health of mothers and babies from routine sources. 

## Methods

### Study design

The data collection and transfer protocol is based on a federated framework model, developed as part of work on European health information systems ^12^ and adopted for research use cases included in the PHIRI project.

The PHIRI project brings together 41 partners in 30 different countries with the aim of sharing data and expertise on the COVID-19 pandemic through a health Information portal on population health and a broader goal of constructing sustainable and reactive health information systems in Europe and promoting their use for policy decisions ^13^. A key component of the project is to conduct research to inform public health policies and management of the COVID-19 pandemic using a federated data model with four use cases, including one on perinatal health and perinatal health inequalities. The perinatal health use case is implemented by the Euro-Peristat network which brings together epidemiologists, statisticians, and clinicians from 31 countries who work with routine data. The network began in 1999 with 10 countries as part of the EU's Health Monitoring Programme, and aims to produce comparable, high-quality data and analysis in reports and scientific publications for use by national, European and international stakeholders who make decisions about the health and health care of pregnant women and babies ^14^.

**Figure 1.**
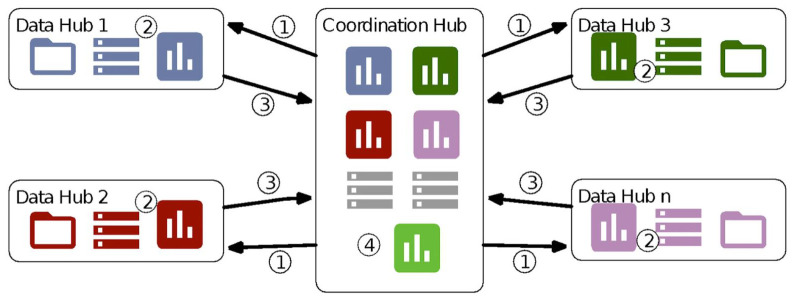
Federated architecture connecting data hubs with the central hub. Numbers in the figure describe the following steps: (1)    Specifications for the data model and R scripts sent to the data hub (2)    Data hub creates CDM according to specifications and runs the R scripts on its own system (3)    Anonymised data tables are sent back to the central hub for analysis (4)    The central hub compiles and analyses these data *NOTE: Annotated figure from Gonzalez-Garcia J, et al. Archives of public health. Dec 9 2021;79(1):221, reprinted with permission*.

In the federated model, individual person-level data (personal data) including outcomes and exposures do not leave systems in the institution with authorisation to hold and analyse them. Instead, anonymised, aggregated data tables and the results of statistical analyses are produced by each institution and then compiled centrally for analysis. The institutions that host and curate data and/or obtain access to individual-level data in accordance with local security and other legislation constitute the data hubs within the federated framework, as illustrated in Figure 1. To implement this model, a common data model (CDM) is specified, defining variables, definitions and formats and the eligible population. These specifications are transferred from the central hub to each of the data hubs (step 1 in the Figure). The CDM is then constructed in each data hub and authorised data controllers within the institutions run open-source R scripts on local servers to produce aggregate tables and statistical results, in terms of means, standard deviations and coefficients from regression models (step 2). After inspection by local analysts, these outputs are transferred to the central hub for synthesis and analysis (step 3 and 4). The data hubs for this study are data providers within the Euro-Peristat network, while the coordinator of the network, at Inserm, is the central hub. Specifications for the common data model and the scripts are stored on the open source depository Zenodo.

### Developing the common data model and scripts: data selection, definitions, and analytic framework

The CDM was based on the Euro-Peristat indicators which are grouped into four themes: fetal, neonatal, and child health, maternal health, population characteristics and risk factors, and health services ^15^. These indicators are collected for all births including stillbirths, live births and terminations of pregnancy – at 22 weeks of gestation and over, or if gestational age is missing, if birthweight is 500 grams or more. The Euro-Peristat indicators are classified as: (1) core indicators that are essential to monitoring perinatal health and are considered highly feasible, and (2) recommended indicators considered desirable for a more complete picture of perinatal health in member countries. The core indicators were automatically included in the CDM, and a three-round on-line consensus process was undertaken to clarify which recommended indicators should be included and whether any new indicators were needed for investigation of the impact of the COVID-19 pandemic.

As a starting point for this consensus process, three lists were developed based on indicators: (1) used in the literature on COVID-19 as determined by a scoping review on the impact of the pandemic on maternal and perinatal health ^16^; (2) proposed in recent systematic reviews of indicators for assessing maternal and newborn care ^17– 19^; and (3) derived from a European survey for women and health care professionals as part of the IMAgiNE EURO project (Improving MAternal Newborn carE in the European Region) ^20^. In total, 44 people from 26 countries participated in the first round of the consensus process, 37 people from 22 countries participated in the second round and 39 people from 29 countries participated in the third round.

Once the indicators and variables were defined, R scripts were programmed to produce the principal indicators for the years 2015 to 2020, following agreed definitions and using table formats previously adopted for Euro-Peristat reports These years were selected to permit assessment of trends over time. Data were also collected by month for key indicators in order to allow analysis of the COVID-19 pandemic in 2020 within specific time windows and to permit use of time series models. The tables and analyses for the project were pre-specified in the project’s protocol, which was finalised before data collection and reviewed by all participants. As part of the development process and in order to reduce the likelihood of errors in the scripts, all scripts were first tested using synthetic databases and then tested with volunteer countries.

### Data collection, cleaning, and validation

Each data hub was responsible for extracting and transforming its data to comply with the CDM and running the R scripts based on common specifications and scripts developed by Inserm in France. Because most data hubs did not have experience using R software, individual on-line meetings were set up by the central hub to provide guidance for the installation of R studio and to run and troubleshoot the scripts. The aim was to correct errors discovered in the files or misunderstanding about definitions and to facilitate the immediate resolution of coding problems and misunderstandings about the CDM.

This protocol includes several data cleaning and validation processes. An initial data check is integrated into the R scripts which provides the number of total and missing observations and basic summary results of the main indicators that are collected. The data provider also reviews the aggregate data tables produced in CSV format before transferring the outputs to the central hub. Several additional steps ensure data quality after tables are provided to the coordination team. The coordination team starts by performing validation checks, including internal validation by verifying the percentage of missing data and consistency between indicators as well as external verification with data collected previously ( 2015 data) and other sources, notably Eurostat. At this stage, queries are sent to the national teams. Second, summary data tables are sent to the data providers from each country for review. Finally, during network meetings, data are presented and compared between countries in order to detect and investigate outliers. Fifteen on-line meetings (11 plenary meetings and four working group meetings) were held to develop the study protocol and to present and discuss the preliminary data. An average of 40 people participated in the plenary meetings and 30 people in the working group meetings.

### Ethics and guidelines for data use and publication

Data were collected in the form of anonymised aggregated data tables or statistical results and therefore do not fall under General Data Protection Regulation which do not apply to anonymised data ^21^. The aggregate tables are designed to be anonymous: each table has no more than three-way cross-tabulations and tables cannot be linked to other tables to augment the number of data items because included items do not overlap. Further, all sociodemographic characteristics, such as age, parity, socioeconomic status, are exported in grouped categories. Aggregate results returned by countries are not subject to cell size limits, unless this is required by the institution’s or the country’s regulations. This is necessary to allow accurate compilation of the indicators. For the publication of results, details on cell sizes under 5 (or 10, if required by the data institution) are not included in reports, web tables or scientific papers. As the study collates routinely collected aggregate data at the country-level and does not involve personal data, an ethical review board was not consulted.

All members of the Euro-Peristat group signed data use and publication guidelines which confirm adherence to the protocol, specify procedures for checking and endorsing data and the rules for authorship of reports and publications using the data.

## Results

### The CDM

The consensus process led to the specification of a core and expanded CDM which produces the indicators included in Table 1. This table also shows which Euro-Peristat indicators were not retained in this process and highlights the indicators relating specifically to the COVID-19 pandemic. All of the Euro-Peristat core indicators, with the exception of maternal mortality, were included in the CDM. Maternal mortality is a rare outcome in Europe (<10/100,000) and therefore not adaptable to a federated approach. In addition, the federated model requires all individual level data to be in the same source, but enhanced data are required to ensure accurate reporting of maternal mortality ^22^. Induction of labour and indicators of socioconomic status, whcih are recommended and new indicators, were also included in the core CDM as these were considered essential for COVID-19 analyses. The expanded CDM produces indicators that focus on healthcare services and utilisation (transfer of the baby to a neonatal intensive care unit or the mother to an adult intensive care unit), length of postpartum stay and level of care and size of the hospital of birth), morbidities (Apgar, maternal pregnancy complications and morbidities), maternal risk factors such as maternal body mass index and breastfeeding.

**Table 1.** Existing Euro-Peristat and new Indicators selected for the PHIRI protocol.

Data category	Core indicators (number ^1^)	Recommended indicators (number)	New indicators
Newborn health outcomes	Stillbirth (C1) Termination of pregnancy (C1) Neonatal death (C2) Infant death (C3) Birth weight (C4) ^2^ Gestational age (C5)	Apgar (R2)	Transfer to NICU Neonatal morbidity For C4: it was decided to modify the definition to include small for gestational age (requires data on sex of baby)
Maternal health outcomes		Maternal morbidity (R5) ^3^ Hysterectomy associated with obstetrical haemorrhage RBC transfusion associated with obstetrical haemorrhage Eclampsia Transfer to ICU	Gestational diabetes Preeclampsia
Population risk factors	Multiple pregnancy (C7) Maternal age (C8) Parity (C9)	Percentage of women who smoke during pregnancy (R8) Distribution of mothers’ education (R9) Distribution of households’ occupational classification (R10) Distribution of mother’s place of birth (R11) Body mass index, BMI (R12)	Socioeconomic (SES) area deprivation score
Health care/medical practices	Mode of delivery (C10) by risk group ^4^	Induction of labour (R15) ^5^ Place of birth (R16) Breastfeeding at birth (R20)	Postpartum hospital stay (mother)
COVID exposures			Date of birth (to be linked to information on infection and societal mitigation measures) COVID infection (ICD or other code) Geographic location (NUTS)
Euro-Peristat indicators not selected for the protocol	Maternal mortality (C6)	Congenital anomalies (R1) Fetal and neonatal deaths due to congenital anomalies (R3) Cerebral palsy (R4) Maternal mortality by cause (R5) Tears to the perineum (R7) Pregnancies following subfertility treatment (R13) Timing of 1 ^st^ prenatal visit (R14) Very preterm infants delivered in units without NICU (R17) Episiotomy (R18) Births without obstetric intervention (R19)	

NOTES 1. Numbers refer to the numbers used by the Euro-Peristat indicators 2. <500g; 500-999g; 1000-1499g; 1500-2499g; 2500-4499; 4500g+; Unknown 3. Changes to the definition were made with individual items being redefined based on the consensus process 4. Multiplicity, Gestational age, Parity, Presentation, Previous Caesarean section 5. Spontaneous onset of labour; induction of labour by medical or surgical means prior to the onset of labour; prelabour caesarean; Unknown

To produce these indicators, the core CDM includes 17 items which are exported in the tables or statistical results and additional variables for running the scripts (time stamps) and allowing verification (id links to original database), while the expanded model includes 18 additional items ( Table 2). The results of the consensus process leading to the choice of these variables and the full data model are presented in the underlying data ^23^.

**Table 2.** Data items included in the Core and Expanded Common Data Models.

*Variable name*	Description
COUNTRY	Country
Year	Year of birth
Month	Month of birth
*Day*	*Day of birth*
*baby_id*	*baby identifier*
*Mother*	*mother identifier*
GA	Gestational age in completed weeks
BW	Birthweight
SEX	Sex of baby
MULT_B	Type of pregnancy (singleton, twin, triplet, or higher order)
VITAL	Vital status at birth (termination of pregnancy, stillbirth, live birth)
NNM	Mortality in first month
NNM_pre	Mortality in first week
IM	Mortality in first year
MATAGE_B	Maternal age at the birth of the baby
PARITY_B	Parity
PRES	Presentation of the baby at delivery
PREVCS	Previous caesarean delivery
MOD	Mode of delivery
TYPECESAR	Type of caesarean (before or during labour)
INSTRUMENT	Instrumental delivery
ONSET	Mode of onset
One socioeconomic (SES) variable (list ordered by preference if several available)	
SES_ED	Educational level of the mother
SES	Deprivation score of area of residence
SES_OccM	Occupation of the mother
SES_OccF	Occupation of the father
Expanded Model	
APGAR	5 minutes Apgar score by gestational age subgroup (preterm, term)
PREPREG_BMI	Mother's prepregnancy body mass index (BMI)
BREASTFED_BIRTH	Breastfeeding at birth
SMOKING	Smoking during pregnancy
COUNTRY OF BIRTH	Maternal country of birth
MAT_MORB_HYST	Severe maternal morbidity (hysterectomy associated with obstetrical haemorrhage)
MAT_MORB_TRANS	Severe maternal morbidity (red blood cell [RBC] transfusion associated with obstetrical haemorrhage)
MAT_MORB_ECLAMPSIA	Severe maternal morbidity (eclampsia)
MAT_MORB_ICU	Severe maternal morbidity (transfer to ICU)
DEL	Volume of annual deliveries of the maternity of birth
NICU_ADM_TERM	Term babies admitted to the neonatal intensive care unit (NICU)
NEONAT_MORB	Neonatal morbidity based on ICD-10 codes
DIAB_PREG	Diabetes in pregnancy
PREECLAMP	Preeclampsia
PPSTAY	Length of postpartum stay
COVID	COVID-19 infection at delivery (use of ICD or other code)
VACCINATION	Whether Covid-19 vaccinations were received
NUTS 2	EU geographic region

NOTE: In blue, used to produce and check the tables, not items used to compute the indicators. In italics, never exported, used for data checking. Full definitions can be found in the CDM: https://doi.org/10.5281/zenodo.6358087

### Feasibility and data availability

During the consensus process, a decision was made to test the core CDM first because of the short timeline of the PHIRI project. In total, 28 countries participated in the implementation and validation of the CDM. In the UK, UK-wide data as well as data from individual nations of the UK were provided (England and Wales combined, Northern Ireland, Scotland, Wales). Table 3 presents the data sources and the data hubs in each country (see Appendix 1 for full lists of data providers and country teams). Twenty-five countries had data for 2020 when data collection was conducted in the spring of 2022. Data for 2020 were provided in the autumn of 2022 for two countries, and Romania provided all data at this time.

**Table 3.** Data hubs participating in the PHIRI protocol test.

	Data sources	Data providers
Austria	* Birth statistics (Statistics Austria) * Cause of death statistics (Statistics Austria)	* Statistics Austria
Belgium	* Vital Statistics, Statistics Belgium (Statbel)	* Statbel
Croatia	* Croatian Medical Birth Database (Croatian Public Health Institute), * Croatian Mortality Database (Croatian Central Bureau of Statistics) -	* Croatian Institute of Public Health
Cyprus	* Medical Birth register (Health Monitoring Unit, Cyprus Ministry of Health) * Causes of Death register (Health Monitoring Unit, Cyprus Ministry of Health) * Database for COVID-19 confirmed cases and deaths (Health Monitoring Unit, Cyprus Ministry of Health)	* Health Monitoring Unit, Ministry of Health
Czech Republic	* Institute of Health Statistics and Information of the Czech Republic (national birth register (mothers and newborns) collecting individual perinatal data.)	* Institute of Health Information and Statistics of the Czech Republic
Denmark	* Medical birth register (The Danish Data authority, Danish Ministry of Health) * National patient register (The Danish Data authority, Danish Ministry of Health) * Danish causes of death register (The Danish Data authority, Danish Ministry of Health) * The Centralized Civil Register	* Statistics Denmark
Estonia	* Estonian Medical Birth Register (National Institute for Public Health) was linked with data from * Estonian Cause of Death Register (National Institute for Public Health)	* Estonian Institute for Population Studies, Tallinn University
Finland	* Medical Birth Register (Finnish Institute for Health Welfare) linked with Central Population Register (Digital and Population Data Services Agency) and Cause of Death Register (Statistics Finland) * Register on Induced Abortions (Finnish Institute for Health Welfare) for late terminations 22–24 weeks	* Finnish Institute for Health and Welfare (THL)
France	* Hospital discharge data ( *Programme de Médicalisation des Systèmes d'Information* (PMSI)) in the French National System of Health Data ( *Système national des données de santé (*SDNS))	* Department for Research, Studies, Assessment and Statistics (DREES), French Ministry of Health
Germany	* IQTIG (Federal Institute for the Quality of Medical Care) * Destatis (Federal Statistical Office)	* IQTIG
Iceland	* The Icelandic Birth Registration * Hospital register (National University Hospital)	* National University Hospital
Ireland	*National Perinatal Reporting System (the Healthcare Pricing Office)	* Healthcare Pricing Office
Italy	* Birth certificates (Ministry of Health) * Causes of deaths (Istat) * Terminations of pregnancies (Istat) * Miscarriages (Istat)	* Italian National Institute for Statistics-ISTAT
Latvia	* Newborn Register of Latvia (Centre for Disease Prevention and Control of Latvia) * Register of Causes of Death (Centre for Disease Prevention and Control of Latvia)	* The Centre for Disease Prevention and Control of Latvia
Lithuania	* Medical Date of Births (Institute of Hygiene Health Information Centre) * Database of the Demographic Statistics (Central Statistical Office) * Causes of Death register (Institute of Hygiene Health Information Centre)	* Institute of Hygiene, Health Information Centre
Luxembourg	* Perinatal Health Monitoring System (Luxembourg Institute of Health) * National Causes of Death Registry (Directorate of Health of Luxembourg)	* Department of Population Health, Luxembourg Institute of Health * Directorate of Health of Luxembourg
Malta	* National Obstetrics Information System (Directorate for Health Information and Research) * National Mortality Register (Directorate for Health Information and Research)	* Directorate for Health Information and Research
Netherlands	* Perined (The Netherlands Perinatal Registry)	* Perined
Norway	* Medical Birth Register of Norway (The Norwegian Institute of Public Health)	* The Norwegian Institute of Public Health
Poland	* Central Statistical Office * Ministry of Health	* National Research Institute of Mother and Child
Portugal	* Instituto Nacional de Estatística – Portugal (Statistics Portugal) * Central Administration of the Health System	* Institute of Public Health of the University of Porto
Romania	* National Institute for Public Health Romania	* National Institute of Public Health Romania
Slovakia	*National Health Information Center	* National Health Information Center
Slovenia	*Perinatal information system (National institute of public health)	* University Medical Centre, Research Unit
Spain	* Vital Statistics (National Statistics Office) * Specialized Care Registry - Minimum Basic Data Set (Ministry of Health)	* Senior Statistical State Corps and Public Health and Addictions Directorate, Generalitat Valenciana
Sweden	* Medical Birth Register (The National Board of Health and Welfare)	* The National Board of Health and Welfare
Switzerland	* Vital Statistics (BEVNAT)	* Swiss Federal Statistical Office
UK, all	* MBRRACE UK (University of Oxford and University of Leicester)	* University of Leicester, MBRRACE-UK collaboration
UK, England, and Wales	*UK, Office for National Statistics (Live birth and stillbirth registration in England and Wales, birth notification in England and Wales)	* Office for National Statistics
UK, Northern Ireland	* Northern Ireland Maternity System - NIMATS	*Public Health Agency (Northern Ireland)
UK, Scotland	* Scottish Morbidity Record 02 (maternity hospital discharge record) * National Records of Scotland Stillbirth, live birth, and infant death registrations (statutory vital event registration)	* Public Health Scotland
UK, Wales	*Digital Health and Care *Wales*	* Digital Health and Care *Wales* ( *DHCW*)

**Figure 2.**
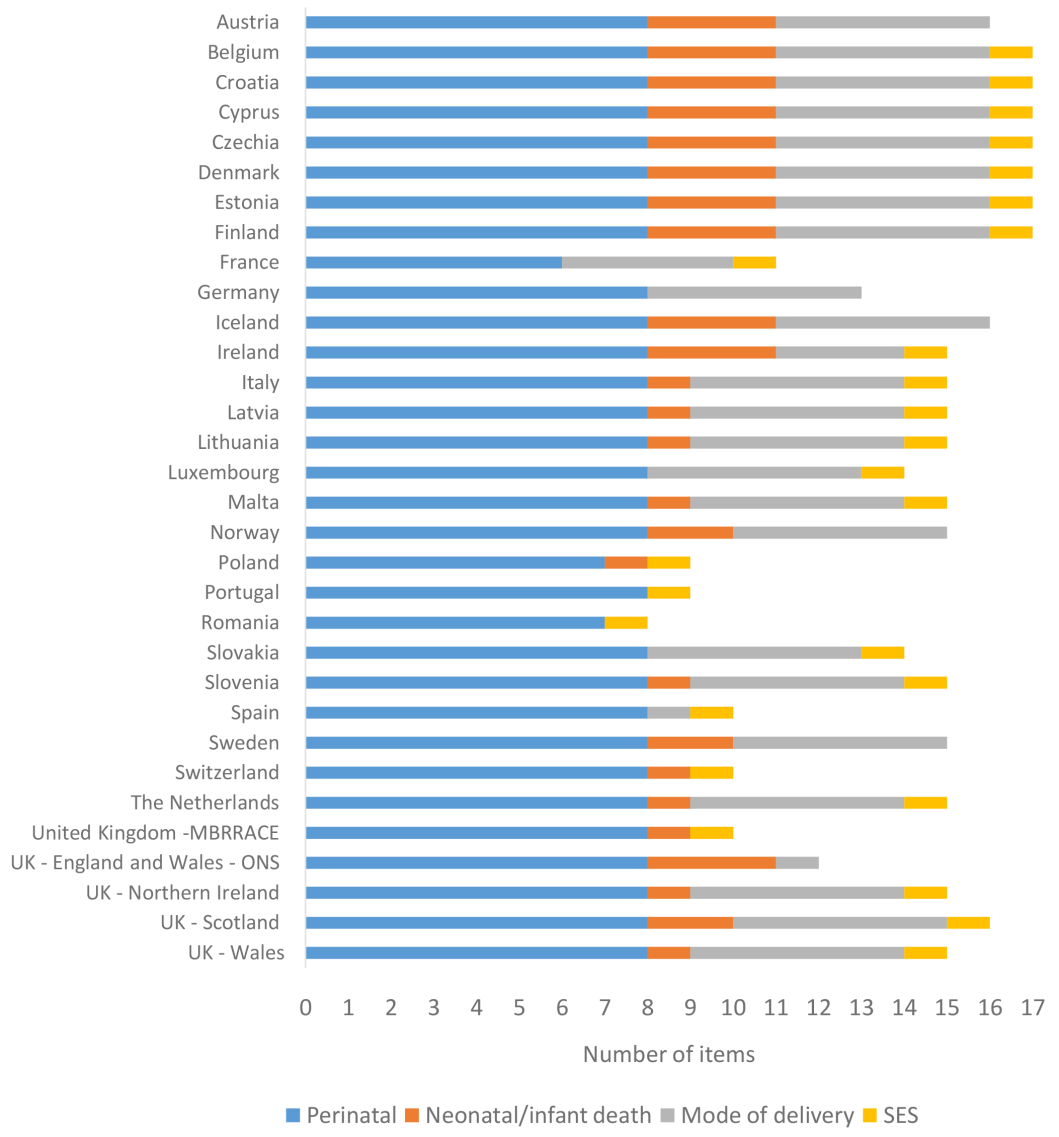
Availability of data for the core common data model by country.

Figure 2 illustrates the availability of data items in the CDM in participating countries, including the nations of the UK. Seven had all 17 required items for the core CDM, while a significant majority had 13 or more items. Six countries had 10 or fewer items. Three types of data items were most likely to be missing: neonatal and infant mortality, mode of birth and induction of labour and a variable for socioeconomic status. For neonatal and infant mortality, these data items are often in different databases which are not linked. Sometimes some data are available, but they are not comprehensive enough for use in surveillance. Further, these deaths can occur in the following year (after birth) and therefore there is a lag for consolidating and merging death data with the corresponding birth data. For mode of birth, some countries use different sources for the surveillance of clinical practices than those used for surveillance of births and deaths.

Socioeconomic data were available in most countries but the variables collected differed, as shown in Table 4. The protocol requested data about mothers’ educational level when this was available. If this data item did not exist, the protocol used area-based deprivation scores or, for countries without deprivation scores, parental occupation. Sixteen countries had data for mothers’ educational level, whereas six countries had area-based deprivation scores and Ireland only collected data on occupation. Seven countries did not have any socioeconomic status data, while Finland had data on maternal occupation, but this variable is incomplete and was not used. Some countries, such as Norway and Sweden, can link socioeconomic data to their birth data, but this is not done routinely.

**Table 4.** Socioeconomic status ^1^ collected according to country.

Mother’s education	Area-based deprivation score	Mother’s occupation ^2^	No data available ^3^
Belgium Croatia Cyprus Czechia Denmark Estonia Italy Latvia Lithuania Luxembourg Malta Poland Portugal Slovakia Slovenia Spain	France Netherlands UK-MBRRACE UK-Northern Ireland UK-Scotland UK-Wales	Ireland	Austria Finland ^4^ Germany Iceland Norway Romania Sweden Switzerland

NOTE: 1. Mother's educational level was the preferred variable, followed by deprivation scores and then parents’ occupation, if several variables were available, 2. No country provided father’s occupation (least preferred indicator); 3. Some countries can link data (i.e. Norway, Sweden), but this is not done routinely; 4. Data on maternal occupation is collected, but missing data are high and this variable is not used.

### Evaluation of the protocol by data hubs

Twenty-five countries responded to the questionnaire about the time needed to implement the protocol. This ranged from 8 hours to 4.5 weeks, as shown in Table 5. The most time-consuming part is the preparation of the dataset for the CDM criteria. This ranged from 4 hours to 4 weeks, depending on whether it was necessary to add data by linking between datasets, for example linking neonatal deaths to live births. Finland reported the shortest time, 4 hours; all information in the CDM is already available in the register and only a short time was needed to format the variables names, recode variables and export the data. In Estonia, the process took longer because of changes in the variable names from year to year, harmonising data on mode of onset of labour and linking infant deaths occurring in the following year. Most of participants needed less than two hours to test the system and less than one hour to run the scripts. Checking data can be a time-consuming procedure, however, ranging from 30 minutes to two weeks. When errors were found, the CDM had to be corrected and the whole set of scripts had to be re-run.

**Table 5.** Time taken and resources needed for implementation and perceived advantages and weaknesses of common data model.

Country (data source)	Data hub has direct access to all data	Time to produce common data model	Time to test system (including installing R)	Time to run R scripts (including repeated runs)	Time to check data	Estimated time to add 2021 data	Estimated time to produce expanded CDM
Austria	Yes	1 day	2 hours	1 hour	4 hours	½ day	0.5 day
Croatia	Yes	12 hours	20 min ^a^	30 min	1 hour	1 hour	8 hours
Cyprus	Yes	A few days	2 hours	<15 minutes	3 hours	A few days	A few days
Estonia	No	6-8 months ^h^	20 min	1 hour	5 hours	6-8 months ^h^	2 days
France	Yes	1 day	1 hour	<15 minutes	1 day	1 day	Some variables are impossible to have. For the others, 1 day.
Finland	Yes	4 hours	2 hours	15 minutes	30 minutes	1 hour	1 hour
Ireland	Yes	2 days	1 day	1 hour		0.5 days	Unsure
Italy	Yes ^f^	3 full days	Couple of days	3 hours		2 full days	Not known yet
Latvia	Yes	few days	2 hours	15-30 minutes		A few days	
Lithuania	Yes	4-5 months	1 hour	3 hours		One month	-
Luxembourg	Yes	3 to 4 days (with 3 people)	0.5 Days	30-60 minutes		A day	0.5 days
Malta	Yes ^f^	2 days	2 hours	3 hours	1-2 days	2 days	1-2 days
Netherlands	Yes	≈one week	NA ^f^	≈3 hours ^b^	≈2 weeks ^c^	2021 not available	
Norway	Yes	≈two weeks (2 people)	Had to install 40 files manually	<15 minutes	≈1 week	2 days	5 days
Poland	Yes	One week	2 hours	2 hours	--	2-3 days	1 month
Portugal	No	3 full days	1 hour	1 hour	3 hours	½ day	Not known yet
Slovakia	Yes	4 days	3 days	1 day	1 day	2 days	Can’t be done
Slovenia	Yes	6 hours	2 hours	<15 minutes	--	2 hours	A few hours
Spain	Yes ^d^	5 days	15 minutes ^e^	<15 minutes ^e^	1 day	2 days	
Sweden	Yes	1 day	Authorised installation required. Estimate:1 to 4 weeks	1 hour	1 hour	1 day to 4 weeks, depending on possible new packages or updates	Not known
Switzerland	Yes	2-3 days	5 days ^g^	2 days	3 days	2 days	Not sure
UK: MBRRACE	Yes	1-2 full days	½ day	4-5 days	--	Probably similar	Probably similar
UK: ONS	Yes	1 week	1 hour ^f^	30-60 minutes	1 day	1-2 days	2-3 days
UK: Scotland	Yes	3-4 weeks	Few hours	5-10minutes	5 days	6-8 weeks	Not sure
UK: Northern Ireland	-	2.5 weeks	1 hour	3 hours	2 days	N/A	-

NOTES: Comments from providers a: but CIPH already has R and we use it also for the other projects b: Scripts worked well. It took a relatively long time is mainly due to the fact that we had to redo it a few times since we just switched to a new dataset and there the process goes different things in the data to came up c: The same applies here that it took a relatively long time (just switched to a new dataset with additional teething problems d: Although data is incomplete as several variables are missing because they are not collected e: R was already installed f: As focal point g: Unfortunately due to difficulties, running even basic R packages at our site h: Ethics committee approval, request to registries, linkage of data i: Does not include the time to create a linked mother/baby cohort from hospital discharge data (project funded by French National Research Agency)

Respondents estimated that adding 2021 data would take between one hour and a few days. The timing of data availability varied from May 2022 to the spring of 2023 ( Table 6). Implementing the expanded CDM was estimated to require between one hour to one month of work, although many respondents were not able to provide an accurate assessment since some of the indicators in the expanded dataset had not previously been collected by Euro-Peristat and checks would have to be made to their coding and conformity to the requested definition.

**Table 6.** Availability of final population birth data for 2021

Country	Timing of availability of finalised data
Austria	July 2022
Croatia	Preliminary data by end of June 2022; Final data by end of October 2022
Cyprus	First trimester of 2023
Denmark	End of May 2022
Estonia	Birth data by the end of April 2022. (Infant deaths in February 2023)
France	Beginning of 2022 (Infants deaths beginning of 2023)
Finland	Preliminary in June 2022; Final data in November 2022
Hungary	By about September 2022
Ireland	Q1 2023
Italy	TOPS and miscarriages: Dec 2022 (final data); Infant deaths: Dec 2023 (final data); Birth certificates Dec 2022 (final data)
Latvia	May 2022
Lithuania	November/December 2022
Luxembourg	September 2022
Netherlands	Q4 2022.
Norway	Most data available in June 2022; Complete data around September 2022
Scotland	October/November 2022
Slovenia	July/August 2022
Spain	Preliminary data in December 2022; Complete data in March 2023
Sweden	December 2022
Switzerland	Mid-July 2022 for civil registration data; maternal health data in November 2022
UK : MBRRACE	Planned December 2022
UK: ONS	Final 2021 births data for England and Wales were first published in August 2022 (this includes stillbirths data), the final 2021 births linked to infant deaths will be published February 2023.

Positive points noted by the participants were the harmonisation of data and the simplicity and efficiency of running the scripts after the dataset has been constructed and tested. As one participant stated “To build this new approach more time is needed, but when it is confirmed it looks better than aggregate excel files. It allows running more detailed analysis. Harmonisation among countries is guaranteed.” Or similarly: “I believe that the data quality is better as everything is the same for all countries and I also think that in the end it will take very little time if we keep doing it this way.” The negative points were the need for a linkage between datasets at an individual level and the time needed to re-check data outputs. As noted: “(This is) more time consuming due to necessity of building cohorts and data linkage of births and infant deaths without an access to the identifier.” The new protocol also is less flexible in taking account of the specific ways in which data are collected within individual countries: “Not all routine national perinatal statistics are collected in the same way in every country. Therefore, a more individualised approach is still necessary in this process.”

## Discussion

This study describes the development and implementation of a federated approach to deriving national perinatal indicators from routine health information systems in Europe. This protocol was based on a set of 17 items for indicators in the core CDM and 18 items for an expanded CDM. The core protocol was successfully implemented in 28 European countries, serving as a successful proof of concept study for a federated perinatal health information system. The main strengths of this approach are the ease of implementation based on open-source methods and R software which can be installed even on secure servers, the use of individual-level data which are held securely on the data controller’s system, ensuring protection of personal data and improvements in comparability resulting from using the same programmes to generate aggregate data tables. Challenges are that data hubs must be able to place all items in the same data file, the substantial time needed to compile the CDM in some countries and the need to rerun the scripts to correct errors or to do additional analyses. Active involvement of data hub participants is essential to ensure effective implementation and maintain data quality.

This federated approach based on a common model with data hubs running R scripts and sending anonymised data tables is simple compared to those used by other research platforms, which deploy software to catalogue or harmonise data on a common data platform and to enable secure exchanges of information. Examples of these are the Obiba suite designed by Maelstrom Research for child cohort platforms in Europe ^24– 27^ or i2b2: Informatics for Integrating Biology & the Bedside used for clinical research collaborations ^28^. More structured architecture for this protocol has been developed by the PHIRI project in the form of a Docker application which is installed in data hubs and houses the CDM and the R scripts to produce outputs. This study did not use this application, which was developed in parallel with the data collection phase, but it has been tested in some countries. This application simplifies the work for some data hubs by providing the analytical environment dealing with dependencies, a graphical user interface providing informative error logs and checks and all the documentation required as a stand-alone application that can be run separately from their systems. On the other hand, it can add to the complexity when working in secure environments where installing customised software is either not allowed or subject to very strict scrutiny.

One feature of this simple federated architecture is that participants must be active at all stages of the process since each set of programmes is run separately and sent to the central hub to be compiled. Other federated models, such as those using the Obiba tools, for instance, set up nodes with harmonised data catalogues and data managers so that an authorised researcher can analyse data from several participating nodes using the statistical software (DataSHIELD) ^29^. This contributes considerable flexibility to the analysis. The simpler model provides a more straightforward guarantee of data protection and safety, however, and ensures that data hubs are involved in decisions about the ways in which data are being extracted and used.

A federated approach also requires good knowledge of the data in each data hub. We benefited from previous work within the Euro-Peristat network to understand national data availability and limitations as well as from analyses to improve comparability of key indicators ^11, 30– 33^. This past experience made it possible to propose harmonised definitions with a script that functioned well for all countries that could implement the approach so far. Implementation of the expanded data model will require more attention to the harmonisation of data, however, because several of the variables have not previously been compiled by the network and some countries will need to use hospital discharge diagnosis and procedure codes which pose challenges for comparisons between countries ^6^.

Despite its simplicity, preparing and testing the model could be time consuming. Data hubs spent between one to two days and several weeks preparing, testing, and checking the data. The personalised approach of one-on-one calls to run the scripts was essential because minor problems, such as formatting issues, use of the wrong code, not having the correct R package, can cause significant delays. Furthermore, another challenge is that any omission or error in the R-scripts means that all countries have to rerun the corrected programmes. This can constitute a major constraint in a network with many participants who are busy and do not have resources specifically allocated to this task. Nonetheless, rerunning of R-scripts and sending updated data is quick, often taking less than 10 minutes once the model and script are working. The project also benefitted from technical support through a help desk provided by PHIRI’s WP7 team for the development of the scripts. In summary, the time to get this system to work is significant and must be integrated into plans for sustainability and expansion.

Although setting up this model is time intensive for some data hubs, it is an improvement on previous procedures used by the Euro-Peristat project for its reports which involved manually outputting multiple tables that were then compiled centrally. By running the scripts using an individual dataset and providing automatic R markdown quality checks and outputs immediately, this approach facilitates harmonisation, leading to better statistics and comparisons, as well as early discovery of errors. In addition, once the system is set up, it can provide a foundation for future work. Adding analyses using the same dataset for all countries or conducting specific sub-studies among interested countries requires only specification of the scripts and rerunning them using the dataset. A final benefit is that this model is designed to be reusable so that other countries or institutions can construct the CDM, run the scripts, and generate tables that can be compared to the Euro-Peristat outputs.

In addition to providing and testing a roadmap for a future information system, this work identified areas where capacity building in terms of data capture or production is required at a national level when countries could not fully implement the protocol. While the expanded data model has yet to be assessed, countries can use the list of items to prioritise health information upgrades. These data items include those needed to compile indicators on healthcare provision and were considered feasible in at least half of participating countries. In many instances, these data exist in databases nationally (or regionally), but they are not brought together into a single database. Countries have resolved these problems through linkage of data from routine sources, which improves the quality and scope of data available for surveillance and research ^34^.

Finally, a common problem is timeliness of data. This significant issue was highlighted by the network in preparatory work for this protocol ^3^ and emerged again in the data-provider survey which showed that final data for 2021 would not be available until the end of 2022 in most countries. The time taken by current processes for producing routine birth data constrains the extent to which it can be used to provide evidence for decision-making. This is especially acute when new infectious disease emergencies arise and data for previous years are no longer relevant. The question of how to speed up the processing of routine data to reduce the time between collection and analysis is a concern in all countries. All the same, our approach, which gave us access to data before official statistics were available and compiled at a European level, could constitute a major step forward in creating a rapid and efficient route between evidence and policy.

A final important point is the quality of the original data, because the effort involved in compiling data is of value only if data are reliable. Therefore, it is important that the methods of data collection are as consistent as possible at an international level and that this is maintained even in emergencies. Integrating other data items that could be used to validate data quality could be explored in future extensions to the CDM.

## Conclusions

This use case focussing on perinatal health illustrates the feasibility of using federated analysis to facilitate rapid production of data and subsequent analysis of key perinatal health indicators in a considerable number of European countries. The successful implementation of this model has implications for future pandemic research and provides a roadmap for developing a routine European health information system to monitor and assess the health of pregnant women and babies.

## Data Availability

Zenodo: PHIRI – WP6 – Use Case C Common Data Model. https://doi.org/10.5281/zenodo.7639001 ^23^ This project contains the following underlying data: protocol EuroPeristat PHIRI-JAN2023.pdf (this document describes the protocol for the Euro-Peristat data collection) UseCaseC_v.1.0.0.zip (this document provides the common data model, with variable names, definitions and formats) UseCaseC_v.2.0.0.zip (this document provides the R scripts needed to produce the aggregated tables and results) Data are available under the terms of the Creative Commons Attribution 4.0 International license (CC-BY 4.0).
