# Supplementary material for: Developing and testing a protocol using a common data model for federated collection and analysis of national perinatal health indicators in Europe
Source: Open Res Eur. 2023 Sep 4;3:54. Originally published 2023 Apr 12. [Version 2] doi: 10.12688/openreseurope.15701.2 (PMC10565425; doi:10.12688/openreseurope.15701.2)
Supplement: Peer review history

# Reviewer report: Reviewer response for version 2

**DOI:** 10.21956/openreseurope.17912.r34904

**Published:** 2023-10-10

**Authors:** Rema Ramakrishnan

**Affiliations:** 1 National Perinatal Epidemiology Unit, University of Oxford, Oxford, England, UK

Thank you for responding to the comments and submitting a revised version of the paper!

 A minor comment:

 It is given that: Further, all sociodemographic characteristics, such as age **, parity**, socioeconomic status, are exported in grouped categories. 

 Parity is not a sociodemographic variable.

Is the rationale for developing the new method (or application) clearly explained?

Yes

Is the description of the method technically sound?

Yes

Are the conclusions about the method and its performance adequately supported by the findings presented in the article?

Yes

If any results are presented, are all the source data underlying the results available to ensure full reproducibility?

Yes

Are sufficient details provided to allow replication of the method development and its use by others?

Yes

Reviewer Expertise:

Perinatal epidemiology, statistics, life-course epidemiology

I confirm that I have read this submission and believe that I have an appropriate level of expertise to confirm that it is of an acceptable scientific standard.

# Reviewer report: Reviewer response for version 2

**DOI:** 10.21956/openreseurope.17912.r34905

**Published:** 2023-09-05

**Authors:** Russell S. Kirby

**Affiliations:** 1 College of Public Health, University of South Florida, Tampa, Florida, USA

I do approve the manuscript with the revisions now made.

Is the rationale for developing the new method (or application) clearly explained?

Yes

Is the description of the method technically sound?

Yes

Are the conclusions about the method and its performance adequately supported by the findings presented in the article?

Yes

If any results are presented, are all the source data underlying the results available to ensure full reproducibility?

No

Are sufficient details provided to allow replication of the method development and its use by others?

Yes

Reviewer Expertise:

NA

I confirm that I have read this submission and believe that I have an appropriate level of expertise to confirm that it is of an acceptable scientific standard.

# Reviewer report: Reviewer response for version 1

**DOI:** 10.21956/openreseurope.16964.r32077

**Published:** 2023-07-19

**Authors:** Rema Ramakrishnan

**Affiliations:** 1 National Perinatal Epidemiology Unit, University of Oxford, Oxford, England, UK

A protocol to enable exchange and federated analysis of data is important not just for researchers in perinatal health but may serve as an exemplar for researchers in other areas too. It is commendable that the authors conducted this study so that perinatal health indicators could be harmonised across countries to enable prompt availability and analysis of data.

 Following are some minor comments/typos:

1. R software: The protocol involved the use of open-source R scripts. Besides this, it is given that ‘By running the scripts using an individual dataset and providing automatic R markdown quality checks and outputs immediately, this approach facilitates harmonisation, leading to better statistics and comparisons, as well as early discovery of errors’. It is unclear how the use of R in contrast to other statistical software such as Stata, SAS, or SPSS can lead to better statistics and comparisons. Usually, the estimates from R, Stata, SAS, or SPSS are identical/similar if the same algorithm is specified. Once the system is set-up, there shouldn’t be much difference in timeliness and quality of output irrespective of the statistical software used. It does seem that some of the data hubs might have been using Excel to compute statistics in which case the above statement may be applicable. Related to this question, it would be informative to provide the number of hubs that did not have experience using R software.
2. ‘These indicators are collected for all births including stillbirths, live births and terminations of pregnancy – at 22 weeks of gestation and over, or if gestational age is missing, if birthweight is 500 grams or more.’ Please clarify the meaning of births included stillbirths, live births, and terminations of pregnancy – termination of pregnancy is in contrast to the definition of birth. In addition to this, the information included in the latter part of the sentence is a bit confusing and contradictory for TOPs.
3. Table 1. Please explain why the population risk factors under the recommended indicators column (Percentage of women who smoke during pregnancy (R8) Distribution of mothers’ education (R9) Distribution of households’ occupational classification (R10) Distribution of mother’s place of birth (R11)) are specific statistics (percentages/distributions) instead of names of indicators.
4. Under feasibility and data availability it is given that in the UK, UK-wide data as well as data from individual nations of the UK were provided (England and Wales combined, Northern Ireland, Scotland, Wales). Does this indicate that data for England are available only in combination with Wales?
5. Table 5. It is difficult to find the location of the superscripts because they are not in the order of occurrence (after a is h, then f, b, c, d,e, g).
6. Table 6. It is given that: UK: MBRRACE: Planned 2022 and UK: ONS the final 2021 births linked to infant deaths will be published February 2023. Should this be changed to current status of data availability?
7. Typo in the spelling of which in the sentence **:** Induction of labour and indicators of socioconomic status, **whcih** are recommended and new indicators, were also included in the core CDM as these were considered essential for COVID-19 analyses.
8. In the abstract there are 31 countries but in the methods section of the main text the number is 30. Which is correct?

Is the rationale for developing the new method (or application) clearly explained?

Yes

Is the description of the method technically sound?

Yes

Are the conclusions about the method and its performance adequately supported by the findings presented in the article?

Yes

If any results are presented, are all the source data underlying the results available to ensure full reproducibility?

Yes

Are sufficient details provided to allow replication of the method development and its use by others?

Yes

Reviewer Expertise:

Perinatal epidemiology, statistics, life-course epidemiology

I confirm that I have read this submission and believe that I have an appropriate level of expertise to confirm that it is of an acceptable scientific standard, however I have significant reservations, as outlined above.

# Reviewer report: Reviewer response for version 1

**DOI:** 10.21956/openreseurope.16964.r31154

**Published:** 2023-05-15

**Authors:** Russell S. Kirby

**Affiliations:** 1 College of Public Health, University of South Florida, Tampa, Florida, USA

This manuscript describes the approach taken to develop a federated analysis of population-based data on births in Europe. The Euro-Peristat network developed a common data model along with program scripts to enable easy preparation and transmission of data on a number of perinatal measures to incorporate into a Europe-wide database. 

 The methods are laid out in detail, and the approach is easy for the reader to follow.

 A few thoughts however. 

- It does not appear that a measure of birth or pregnancy interval is included. This might be added as an indicator for future development.
- It might also be useful to include maternal rehospitalization within 14 days of discharge.
- Also, consideration for how each contributing register accesses area-based measures and quantifies these would be helpful. There are several SES measures for example, some of which are based on the mother/father and others area-based. If the register links out to population-based census data for small areas, this might facilitate a broader range in inquiry concerning the contribution of life course or socio-ecological factors.

All in all, this appears to be an excellent example of international cooperation to build a data structure that serves the broader needs of the Europe region which also supporting the work of the member registries.

Is the rationale for developing the new method (or application) clearly explained?

Yes

Is the description of the method technically sound?

Yes

Are the conclusions about the method and its performance adequately supported by the findings presented in the article?

Yes

If any results are presented, are all the source data underlying the results available to ensure full reproducibility?

No

Are sufficient details provided to allow replication of the method development and its use by others?

Yes

Reviewer Expertise:

NA

I confirm that I have read this submission and believe that I have an appropriate level of expertise to confirm that it is of an acceptable scientific standard.
